# Reproductive Late Effects in Female Survivors of Childhood Cancer

**DOI:** 10.1155/2012/564794

**Published:** 2012-05-06

**Authors:** Shivany Gnaneswaran, Rebecca Deans, Richard J. Cohn

**Affiliations:** ^1^University of New South Wales, Sydney, NSW 2052, Australia; ^2^Royal Hospital for Women, Barker Street, Randwick, NSW 2031, Australia; ^3^Centre for Children's Cancer and Blood Disorders, Sydney Children's Hospital, High Street, Randwick, NSW 2031, Australia

## Abstract

Childhood cancer treatments can cause female reproductive late effects. Radiation to the hypothalamic-pituitary-ovarian axis is associated with altered menarche, miscarriage, and implantation failure. Patients who receive chemotherapy and/or ovarian radiation are at risk of premature ovarian failure; the risk increases with increasing radiation dose, alkylating agent score, combination therapy, and older age at treatment. Ovarian reserve may be assessed using antimullerian hormone assay and ultrasound measurements of ovarian volume and antral follicle count; however, their efficacy is poorly established in this cohort. Fertility preservation options including cryopreservation, oophoropexy, and gonadotropin-releasing hormone analogues may be initiated prior to treatment, although most are still considered experimental. Uterine radiation has been linked to pregnancy complications including miscarriage, premature delivery, stillbirth, low-birth-weight and small-for-gestational-age infants. This paper summarises the literature on female reproductive late effects. The information should facilitate counseling and management of female survivors throughout their reproductive lives.

## 1. Introduction

Cancer is the second commonest cause of death in children in developed countries [1]. Common childhood cancers include leukaemia, lymphoma, rhabdomyosarcoma, neuroblastoma, Wilms' tumour, central nervous system tumours, and germ cell tumours [2, 3]. Most of these cancers are curable using chemotherapy, radiotherapy, or surgery, either alone or in combination [2, 4]. More aggressive or treatment-refractory cancers require intensive multimodal therapies involving multiagent chemotherapy [4].

As a result of advances in paediatric cancer treatment protocols, survival rates from childhood cancers have improved dramatically over the past 3 decades [2]. The expected 5-year survival rate for newly diagnosed patients is at least 70% [2, 5].

Despite being highly successful in treating cancers, therapies such as chemotherapy and radiotherapy have also produced complications referred to as late effects [6]. Late effects can either arise during treatment or shortly thereafter to persist as chronic conditions. They may also manifest years after the completion of therapy [4]. Late effects encompass a range of clinical conditions including neurocognitive deficits, skeletal deformities, cardiopulmonary, and renal and hepatic damage, as well as endocrine and reproductive dysfunction. It is estimated that 60–75% of survivors of childhood cancer will develop at least one late effect as a direct result of their treatment [7].

The female reproductive system is especially vulnerable to late effects of cancer therapy. Normal hypothalamic, pituitary, ovarian, and uterine functions as well as adequate ovarian reserve are required for pubertal progression, fertility and pregnancy [2]. Potential late effects on the female reproductive system can therefore occur as a result of chemotherapy and/or radiation to the hypothalamic-pituitary-ovarian (HPO) axis, ovaries or uterus [2, 4]. Although the late effects show individual variation, there is a strong relationship with the treatment received [2].

The purpose of this paper is to summarise the literature regarding the influence of childhood cancer therapies on female reproductive late effects, measures to assess ovarian reserve, and options for fertility preservation. Literature was obtained from electronic resources including Medline, Embase, PubMed Central, Journals@Ovid, and The Cochrane Library (including The Cochrane Database Of Systematic Reviews). The Medical Subject Headings (MeSH) and keywords “childhood cancer,” “pediatrics/child/paediatric,” “neoplasms/malignancy,” “menarche,” “menopause/premature menopause/amenorrhea/female infertility/ovarian failure,” “pregnancy/pregnancy complications/pregnancy outcome,” “fertility preservation,” “GnRH analogues/GnRH agonist,” “ovarian tissue cryopreservation,” “embryo freezing/embryo cryopreservation,” and “oocyte cryopreservation” were used. Relevant references cited by the obtained literature were also acquired independently.

## 2. Late Effects of Radiotherapy to the Hypothalamic-Pituitary-Ovarian Axis

Children who receive radiation to the brain are at risk of damage to the hypothalamus and pituitary with subsequent changes in the release of pituitary gonadotropins to stimulate the ovaries [8].

Earlier timing of menarche has an association with cranial radiation. Low dose radiation (18–24 Gy), as part of treatment for acute lymphoblastic leukaemia (ALL), and early menarche has been reported in previous studies [9–14]. Currently, although many ALL treatment protocols favour more intensive alkylating agent chemotherapy, 10–15% of patients continue to receive cranial radiation upfront [15]. Within this cohort there remains a link with early menarche; however, there is no increased risk amongst patients treated for ALL with chemotherapy only [15]. Patients treated with higher doses of radiation for CNS tumours are also at risk of an earlier menarche, with one study of 235 survivors exposed to cranial radiation prior to menarche determining doses >50 Gy to be a significant independent risk factor [16]. Furthermore, a younger age at cranial radiation has been independently associated with early menarche in patients treated for ALL and CNS tumours (<5 years and <4 years, resp.) [15, 16].

Patients who receive radiation to the lumbar-sacral spine have an increased risk of delayed menarche [11–13, 15]. This is presumably due to indirect radiation effects on the ovaries. However, as some of these patients also receive radiation to the brain, it is possible that gonadotropin deficiency may contribute to their delayed menarche. Radiation doses >50 Gy to the hypothalamus/pituitary in combination with spinal radiation have been reported to increase the risk of delayed menarche 12-fold compared to patients who do not receive radiation [16]. Although alkylating agent exposure is known to increase the risk of gonadal damage, no such associations have been noted to date.

Lower pregnancy rates have been described in patients who received cranial radiation, although the radiation doses reported to decrease the risk of a pregnancy are variable. In one study of 5149 female survivors [17] radiation doses >30 Gy to the hypothalamus/pituitary were a significant risk factor for not having a pregnancy, whilst in another study [18] a decreased risk of pregnancy was noted in patients receiving >22 Gy to the hypothalamus/pituitary. Lower dose exposures (18–24 Gy) used in the treatment of ALL have also been shown to decrease fertility rates in female survivors compared to sibling controls [19, 20], particularly in patients who receive 18–24 Gy to the brain within two years of menarche and whose age at first pregnancy is 18–21 years [19]. Further studies of proven fertility are limited to clarify these findings; however, Bath et al. [21] have observed decreased luteinizing hormone (LH) excretion, decreased LH surge, and high frequency of short (≤11 days) luteal phase (despite regular (26–30 day) ovulatory menstrual cycles) in 12 ALL survivors treated with 18–24 Gy cranial irradiation. These findings could suggest subnormal mid-cycle LH surge and decreased progesterone production by the corpus luteum as one of the causal factors for delayed endometrial maturation and subsequent implantation failure and/or infertility [18].

It has been suggested that cranial radiation increases the risk of miscarriage (<24 weeks) possibly through impairment of HPO axis function [22]. Two studies have shown some support for this hypothesis reporting 1.8- and 1.4-fold significantly increased risks, respectively, amongst survivors treated with cranial radiation only [23, 24]. Another study reported an increased risk amongst patients treated with cranial and craniospinal radiation; however, there was no distinction in the risk of first-trimester miscarriage (<12 weeks) between these treatment groups [25]. 

## 3. Late Effects of Treatment to the Ovaries

There is a relationship between age and the number of primordial follicles in human ovaries [2]. At 5-6 months gestation, the number of follicles reaches a maximum of approximately 7 × 10^6^. Thereafter, there is an exponential decline with approximately 400 follicles released as mature oocytes during the reproductive lifespan. Accelerated decline in follicle number occurs after 35 years until menopause occurs. This occurs at an average age of 50.4 years in the Western world [2, 26–30].

Any radiation or chemotherapy will deplete the number of follicles and induce damage to the ovaries [2]. Patients who are older at the time of either treatment have an increased risk of ovarian damage as there is a greater reserve of primordial follicles in younger patients [2, 25, 28]. It has also been suggested that the oocytes present in older patients are more vulnerable to gonadal toxins [31, 32]. Accordingly the mean sterilising dose of radiation to the ovary at 12 years of age has been estimated at 18 Gy compared to 9.5 Gy at 45 years of age [33].

## 4. Direct Radiotherapy to the Ovaries

Additional to scatter from lumbar-sacral radiation, the ovaries can also be irradiated directly as part of abdominal, pelvic, or total body irradiation. Premature ovarian failure may take the form of either acute ovarian failure (AOF), where there is a loss of ovarian function during or shortly after the completion of cancer therapy, or premature menopause, defined as menopause younger than 40 years in survivors who retain ovarian function following treatment [28, 33]. The specific risk of premature ovarian failure after direct radiation to the ovaries is site and dose-dependent [28, 34]. Stillman et al. [35] reported ovarian failure in none of 34 survivors who had both ovaries outside of the field of abdominal radiation, in 14% of 35 survivors whose ovaries were at the edge of the radiation field, and in 68% of 25 survivors who had both ovaries entirely within the field of irradiation. Previous studies have demonstrated ovarian doses >10 Gy to be linked to a high risk of AOF, especially doses >20 Gy which are associated with the highest rate, with over 70% of a study cohort of 3390 survivors developing AOF [34]. Recently, doses as low as 5 Gy to the ovaries have been identified as a significant risk factor for not having a pregnancy, presumably due to ovarian failure [17]. The LD_50_ (the radiation dose required to kill 50% of oocytes) of the human oocyte has been estimated at <2 Gy [25, 28].

## 5. Chemotherapy

Factors affecting the risk of ovarian injury in children treated with chemotherapy include the specific agent, the number of agents, and the cumulative dose [4]. Several chemotherapeutic agents when given at high doses are recognised as toxic to young ovaries including alkylating agents, cisplatin procarbazine, and the nitrosoureas (CCNU and BCNU) [2, 36]. There is currently no data on threshold doses to cause ovarian failure, although it has been noted that exposures to procarbazine at any age or cyclophosphamide between 13 and 20 years are independent risk factors for AOF [34]. Additionally, cyclophosphamide and CCNU have been associated with a lower risk of pregnancy, with fertility rates decreasing with increasing doses of these agents [17]. Increasing alkylating agent score (based on the number of alkylating agents and cumulative doses) has also been identified as a risk factor for both nonsurgical premature menopause [34] and decreased fertility, with one study of 5149 survivors demonstrating that alkylating agent scores of three and four were associated with a lower observed risk of pregnancy compared to patients who had no alkylating agent exposure [17].

## 6. Combination Radiotherapy and Chemotherapy

A combination of radiation to the ovaries and chemotherapy poses the greatest risk of ovarian failure [25, 28, 37–39]. Amongst patients who are treated with alkylating agents plus abdominopelvic irradiation, 30–40% are estimated to develop non-surgical premature menopause [37, 39].

## 7. Assessment of Ovarian Reserve

### 7.1. Hormonal Markers

A slow and steady compensatory rise in early follicular phase follicle-stimulating hormone (FSH) has traditionally been used as marker of perimenopause and ovarian reserve [28, 40]. Unless significantly elevated however, early follicular phase FSH as an isolated test is not a sensitive early marker of diminished ovarian reserve [28]. Some women may experience transiently elevated FSH unrelated to their pool of follicles, which can add to potential erroneous presumptions of premature menopause. Inhibin-B is another hormonal marker which has been proposed to assess ovarian reserve. It is produced by follicles following recruitment during the early follicular phase and has been shown to decrease with age and during premature ovarian failure [40, 41]. However, it is a fairly late marker of reduced follicle reserve as levels do not decrease gradually with age [40].

Levels of antimullerian hormone (AMH) are more reflective of the number of preantral follicles and are thus a marker of oocyte pool [28, 40, 42, 43]. AMH levels in women of reproductive age appear to have a greater sensitivity and specificity for ovarian reserve over FSH and inhibin-B [40, 44]. Levels are independent of the phase of the ovarian cycle and should not vary significantly between menstrual cycles as levels are not dependent on the feedback mechanisms of the HPO axis [28, 40, 42–44]. Importantly, AMH levels decrease steadily over time and often fall before other markers of ovarian ageing occur [42]. There is a demonstrated age-dependent decrease after 30 years, with a decline in AMH levels below 0.086 *μ*g/L signalling menopause [45]. Although a promising marker for ovarian reserve, its efficacy in assessing premature menopause and chance of pregnancy in young patients after cancer treatment is not well established, and more data are required.

### 7.2. Ultrasonographic Markers

Transvaginal ultrasound assessments of total ovarian volume and antral follicle count (AFC) are noninvasive and accurate tests of ovarian reserve as both exhibit an age-related decline [28, 40]. Mean premenopausal ovarian volumes of 4.9 cm^3^ compared to mean postmenopausal volumes of 2.2 cm^3^ have been determined [46]. The mean AFC is 15 at 25–34 years of age and decreases to 4 at 41–46 years of age [47]. AFC has also been shown to correlate tightly with plasma levels of AMH [48].

## 8. Options for Fertility Preservation

There are two main approaches to preserving fertility in female childhood cancer survivors, namely, cryopreservation of ovarian tissue, oocytes, and embryos, and interventions to minimise the effects of cancer therapies on the ovaries [49]. Within these approaches, there are established practices and experimental strategies.

### 8.1. Cryopreservation

Embryo cryopreservation is the main established method of fertility preservation, with delivery rates per embryo transfer ranging between 10–40% depending upon the age of the female partner and quality of oocyte [49, 50]. However, this option is of limited value in children as the patient must be postpubertal and have a partner or use donor sperm. This process also requires at least one cycle of ovarian stimulation which may not be possible when chemotherapy needs to be commenced immediately or where stimulation is contraindicated due to hormone-sensitive tumours [49, 51, 52].

In contrast, oocyte cryopreservation may be utilised in some adolescent girls as it does not require partner or donor sperm. However, the method also requires the use of ovarian stimulation [53] and its success is dependent on the total number of oocytes retrieved (<10 oocytes is associated with minimal chance of pregnancy), which is often difficult in sexually immature patients [54, 55]. Ongoing advances in oocyte cryopreservation technique and the use of intracytoplasmic sperm injection (ICSI) appear to have improved success rates [53, 54]. In a prospective randomised controlled trial (RCT) conducted by Smith et al. [56], oocyte survival, fertilization, and establishment of pregnancy were significantly higher following vitrification/warming compared with freezing/thawing. In another RCT [57] fertilisation and embryo development rates using vitrified oocytes followed by ICSI approached that of fresh oocytes after ICSI. Cryopreservation of oocytes has also been described in association with ovarian tissue cryopreservation (OTC) in prepubertal girls, whereby any antral follicles observed on the ovarian surface at the time of biopsy are aspirated, matured in vitro and cryopreserved [55, 58]. Accordingly, Revel et al. [58] were able to cryopreserve 11 mature oocytes from three prepubertal girls aged 5, 8, and 10 years.

Ovarian tissue cryopreservation is the only means of preserving fertility in prepubertal girls [49, 51, 53, 59] and may also be utilised in girls who do not have enough time to undergo ovarian stimulation for oocyte and embryo cryopreservation [49, 51, 53, 57]. Ovarian cortical tissue is harvested laparoscopically without preparation, cryopreserved using standard slow-programmed freezing, and is reimplanted into the pelvic cavity (orthoptic site) or a heterotopic site once the patient is in remission [51]. The option has greater fertility potential in prepubertal girls due to a greater density of primordial follicles in the harvested tissue [54, 55]. This process has the added advantage of endogenous hormone production by the ovarian tissue, and avoidance of hormone therapy for bone health maintenance. To date, ten live births have been reported following orthoptic reimplantation of frozen-thawed ovarian cortex harvested from postpubertal girls [55], although the origins of these pregnancies are not definite as a vast majority of patients have demonstrated restoration of follicular growth and ovulation [55]. Risks of OTC include the surgical risks associated with the invasive procedure. An additional concern is reimplantation of the primary tumour and/or malignant transformation of reimplanted tissue, which is possible with leukaemias, neuroblastoma, and Burkitt's lymphoma which are common during childhood and metastasise to the ovaries [49, 53], although histological analysis of the cryopreserved ovarian cortex and further methods for monitoring minimal residual disease have been developed over recent years [55].

### 8.2. Interventions to Minimise Damage Caused by Cancer Therapies

Transposition of the ovaries (oophoropexy) outside of the field of radiation may be performed to reduce the ovarian radiation dose to 5–10% of that if the ovaries remained in situ [60]. The ovaries can either be relocated outside of the pelvis, in the case of pelvic irradiation, or, in the case of craniospinal irradiation, fixed laterally as far as possible from the spine [55]. Preserved ovarian function following oophoropexy outside of the pelvis has been reported between 16–90% [49, 54]; the wide variability is due to the inability to calculate and prevent scatter, combination chemotherapy and different radiation doses [49, 54]. A disadvantage of this technique is the invasive procedure which needs to occur at a time when the patient is planning cancer treatment. Moreover, ovarian failure may ensue if the ovaries are not transposed far enough or if they revert back to their original position or if the vascular supply of the ovary is affected by the surgical procedure [49]. Additional issues to consider prior to performing extrapelvic oophoropexy include problems achieving a spontaneous pregnancy and difficulties with oocyte retrieval for in vitro fertilisation (IVF) unless a second procedure is performed to relocate the ovaries back to the pelvis [49, 53]; this problem is often avoided in the case of lateral oophoropexy for cranial irradiation as the anatomic relations of the ovary with the uterus and fallopian tubes are maintained [55]. Commonly, ovarian biopsy and transposition is performed simultaneously for patients undergoing combination therapies.

GnRH (gonadotropin-releasing hormone) analogues have been suggested as chemoprotective agents. The exact mechanism remains unclear, although it is hypothesised that suppression of pituitary gonadotropin production with subsequent reductions in ovarian follicular cell division and growth render the follicles less vulnerable to cytotoxic agents [49]. Their use is limited in prepubertal girls who are hypogonadal. To date, the evidence for the use of GnRH analogues is controversial. There are several case series and small cohort studies that claim benefit [61–63], and a meta-analysis has shown benefit in this technique; however, when only high quality studies are included, the results are not significant [64]. A recent randomised study of 49 breast cancer patients with 30-month followup additionally found no difference in the incidence of ovarian failure [65]; however, a prospective multicentre study is currently underway with anticipated results.

## 9. Late Effects of Radiotherapy to the Uterus

Radiation to the uterus can impair uterine function, causing reduced uterine volume, decreased myometrial elasticity, and some uterine vascular damage [4, 25, 66–68]. Although data on threshold doses for uterine dysfunction is limited, earlier studies have reported reduced uterine length, poor endometrial thickness in response to oestradiol therapy, and absence of uterine artery blood flow detectable by Doppler ultrasound in patients treated with 14–30 Gy of radiation to the uterus [67, 69, 70]. The risk of uterine dysfunction increases with higher radiation doses and fields involving a greater uterine volume [4]. Radiation prior to puberty has also been associated with irreversible damage to the uterus, with prepubertal uterine morphology observed in postpubertal patients [68].

Reduced adult uterine volume and blood supply may restrict foetal growth and the ability to carry the foetus to term [22]. There is an increased risk of delivering low birth weight infants (<2.5 kg) amongst patients treated with abdominal/pelvic radiation [22, 23, 25, 71–73]. Patients who receive >5 Gy radiation to the uterus are also significantly more likely to deliver small for gestational age offspring (<10th percentile for gestational age) [25]. Higher frequencies of preterm birth (<37 weeks) following abdominal/pelvic radiation have additionally been reported [25, 71, 72, 74] with a more recent study observing a 2-fold elevated risk of preterm delivery in their cohort of 351 survivors who received abdominal radiation [22]. There is also an increased risk of miscarriage [22–24], with Reulen et al. reporting the risk to be particularly elevated during the second trimester [22].

In one study of 39 patients who received radiation to the pelvis a significantly increased risk of stillbirth at doses >10 Gy was observed [75]. Additionally, doses as low as 1.0–2.49 Gy were observed to significantly increase the risk in girls treated before menarche [75]. Although the exact mechanism of decreased uterine volume and blood supply on stillbirth is unknown, it is possible that these effects may increase the risk of placental or umbilical cord anomalies [75].

## 10. Conclusion

Although there is individual susceptibility, the late effects of childhood cancer therapies on the reproductive system can be anticipated amongst female childhood cancer survivors throughout their reproductive lives. Girls treated with radiation to the HPO axis are at risk of abnormal timing of menarche and pregnancy sequelae including miscarriage and implantation failure contributing to infertility. Survivors treated with chemotherapy and/or radiotherapy affecting ovarian reserve are also at risk of premature ovarian failure. Women with uterine dysfunction following radiotherapy are at risk of pregnancy complications including miscarriage, low-birth-weight and small-for-gestational-age infants, premature delivery, and stillbirth.

These findings have important implications on counseling and management. Girls and their families should be counseled regarding options for fertility preservation, the possibility of abnormal pubertal progression and menstrual dysfunction. Women who are at risk of premature ovarian failure should be advised to not delay their childbearing, have assessment of ovarian reserve with referral for specialist fertility consultation as required. Pregnant survivors who have had radiation to the uterus should be managed in a high-risk obstetric unit.
